# Learning and Memory Impairments in Patients with Minimal Hepatic Encephalopathy are Associated with Structural and Functional Connectivity Alterations in Hippocampus

**DOI:** 10.1038/s41598-018-27978-x

**Published:** 2018-06-25

**Authors:** Raquel García-García, Álvaro Javier Cruz-Gómez, Amparo Urios, Alba Mangas-Losada, Cristina Forn, Desamparados Escudero-García, Elena Kosenko, Isidro Torregrosa, Joan Tosca, Remedios Giner-Durán, Miguel Angel Serra, César Avila, Vicente Belloch, Vicente Felipo, Carmina Montoliu

**Affiliations:** 1 Laboratory of Neurobiology, Centro Investigación Príncipe Felipe, Valencia, Spain; 2 Departamento Psicologia Basica, Clinica y Psicobiologia. Universitat Jaume I, Castellon, Spain; 3 Fundacion Investigacion Hospital Clinico Valencia. INCLIVA, Valencia, Spain; 4 Unidad de Digestivo-Hospital Clínico. Departamento Medicina, Universidad Valencia, Valencia, Spain; 5 Institute of Theoretical and Experimental Biophysics, Pushchino, Russia; 6 Servicio Nefrologia. Hospital Clinico Valencia, Valencia, Spain; 7 Servicio Digestivo. Hospital Arnau Vilanova, Valencia, Spain; 8 ERESA, Unidad de RM, Valencia, Spain; 9 Departamento Patología, Facultad Medicina, Universidad Valencia, Valencia, Spain

## Abstract

Patients with minimal hepatic encephalopathy (MHE) show mild cognitive impairment associated with alterations in attentional and executive networks. There are no studies evaluating the relationship between memory in MHE and structural and functional connectivity (FC) changes in the hippocampal system. This study aimed to evaluate verbal learning and long-term memory in cirrhotic patients with (C-MHE) and without MHE (C-NMHE) and healthy controls. We assessed the relationship between alterations in memory and the structural integrity and FC of the hippocampal system. C-MHE patients showed impairments in learning, long-term memory, and recognition, compared to C-NMHE patients and controls. Cirrhotic patients showed reduced fimbria volume compared to controls. Larger volumes in hippocampus subfields were related to better memory performance in C-NMHE patients and controls. C-MHE patients presented lower FC between the L-presubiculum and L-precuneus than C-NMHE patients. Compared to controls, C-MHE patients had reduced FC between L-presubiculum and subiculum seeds and bilateral precuneus, which correlated with cognitive impairment and memory performance. Alterations in the FC of the hippocampal system could contribute to learning and long-term memory impairments in C-MHE patients. This study demonstrates the association between alterations in learning and long-term memory and structural and FC disturbances in hippocampal structures in cirrhotic patients.

## Introduction

Hepatic encephalopathy (HE) is a complex neuropsychiatric syndrome due to impaired central nervous system function associated with liver cirrhosis^1,2^. About 33–55% of cirrhotic patients without obvious clinical symptoms of HE show minimal hepatic encephalopathy (MHE) with mild cognitive impairment. MHE patients present attention deficits, visuo-motor and coordination disorders, and impairments in visual perception, visual orientation and visuo-constructive abilities^3–8^. However, although many studies have evaluated the memory of these patients, the results are contradictory and do not clearly determine whether or not there is a memory alteration in MHE^9–16^.

The processes of learning, memory, and cognition are related to the hippocampus^17,18^. This region has widespread connectivity, and hippocampal lesions that include damage to the anterior hippocampus have a deleterious effect on learning, memory, and navigation^19,20^. The hippocampus is not a unitary anatomical formation. Several subfields have been identified, that differ in both their histological grounds^21^ and their efferent connections^22^. Functional neuroimaging studies have demonstrated a relationship between high-level cognitive functions and specific subfields within the hippocampus^18,22^.

Magnetic resonance imaging (MRI) makes it possible to study the neuroanatomical and functional changes associated with pathologies such as MHE, and associate these changes with specific cognitive deficits, such as alterations in memory. A significant decrease in gray matter density in several brain areas has been described in cirrhotic patients^23,24^. In previous studies, we found a reduction in hippocampus volume in MHE patients compared to healthy controls^24,25^. Moreover, MHE patients presented focal damage in the precuneus (involved in the Default Mode network, DMN) and alterations in white matter microstructural integrity, which correlate with cognitive alterations^25,26^.

Recent studies have assessed the relationship between cognitive impairment associated with MHE and measures of changes in functional connectivity (FC) patterns in resting state^24,27–33^. These studies focus on investigating the extent to which different brain regions are connected and analyzing their joint activity or temporal synchronization. Relevant functional networks, such as the DMN and attention networks, the visual network, and thalamo-cortical circuits, are disturbed in cirrhotic patients^27–34^. Patients with MHE show a significant decrease in gray matter volume and reduced functional connectivity in different networks related to attention and executive functions, compared to controls and patients without MHE^24,27–29,33^.

Several studies support the idea of an interplay between hyperammonemia and inflammation in the neurological alterations in MHE^35,36^. Moreover, the levels of serum pro-inflammatory cytokines interleukin-6 (IL-6) and interleukin-18 (IL-18) correlate with the presence of MHE^37^.

There are no studies that have evaluated the structural and FC changes of the different hippocampal subfields in relation to memory function, cognitive performance, and markers of inflammation in cirrhotic patients with MHE.

The aim of this study was to evaluate the memory processes of learning, long-term memory, and recognition in patients without and with MHE and in healthy controls. We assessed the relationship between memory function and alterations in the structural integrity and functional connectivity of the different subfields in the hippocampal system by means of magnetic resonance techniques. We also assessed the correlations among ammonia and inflammatory markers, memory function, and magnetic resonance parameters.

## Results

### Demographic, clinical, and neuropsychological results

Table 1 summarizes the sociodemographic, clinical, and neuropsychological data of the experimental groups included in the study: 38 patients with liver cirrhosis and 24 healthy controls without liver disease. Cirrhotic patients were classified as without (C-NMHE) or with (C-MHE) mild cognitive impairment (i.e. MHE), according to the Psychometric Hepatic Encephalopathy Score (PHES) (see Materials and Methods section). Groups did not differ on age. Regarding CVLT performance, cirrhotic patients with (C-MHE) and without MHE (C-NMHE) showed significant differences, compared to controls on learning trials and the total learning score. C-MHE patients learned significantly fewer words than C-NMHE patients from trials 3 to 5. Total learning, delayed recall, and recognition scores were also significantly different in C-MHE patients, compared to C-NMHE (p < 0.01, p < 0.05 and p < 0.01, respectively) and controls (p < 0.001, p < 0.001 and p < 0.01, respectively) (Table 1).

**Table 1:** Main demographic, clinical and neuropsychological characteristics of all participants.

	Controls (n = 24)	C-NMHE patients (n = 25)	C-MHE patients (n = 13)	C-NMHE *P vs*. controls	C-MHE *P vs*. controls	C-MHE *P vs*. C-NMHE
Age (range)	60 ± 1 (50–73)	63 ± 2 (50–81)	64 ± 3 (49–85)	0.863	0.419	1.000
Gender (Male/Female)	16/8	19/6	12/1			
Child Pugh A/B/C	—	22/3/0	8/5/0			
Alcohol/HCV/ others	—	11/11/3	6/3/4			
PHES score	0.9 ± 0.2	−0.9 ± 0.3	−7.3 ± 0.9	**0.003**	**<0.001**	**<0.001**
**CVLT** ^**a**^						
*Learning trial 1*	6.2 ± 0.3	4.7 ± 0.5	4.1 ± 0.3	0.042	**0.012**	1.000
*Learning trial 2*	9.1 ±0.3	7.3 ± 0.6	6.2 ± 0.5	0.039	**0.003**	0.572
*Learning trial 3*	10.9 ± 0.5	8.5 ± 0.6	6.2 ± 0.7	**0.009**	**<0.001**	0.047
*Learning trial 4*	11.7 ± 0.5	9.5 ± 0.6	6.6 ± 0.7	**0.009**	**<0.001**	0.005
*Learning trial 5*	12.7 ± 0.4	10.7 ± 0.7	6.8 ± 0.8	0.05	**<0.001**	0.001
*Total Learning*	51 ± 1	41 ± 3	30 ± 3	**0.004**	**<0.001**	0.011
*Delayed Recall*	11.7 ± 0.5	9.7 ± 0.8	7.1 ± 0.8	0.110	**<0.001**	0.013
*Recognition*	14.9 ± 0.3	15 ± 0.2	13.3 ± 0.5	1.000	**0.008**	0.005
***Biochemical determinations***						
Blood ammonia (μM)	9 ± 0.7	25 ± 5	36 ± 7	0.023	0.001	0.357
Plasma cGMP (pmol/ml)	4.2 ± 0.3	8.4 ± 1.2	13 ± 1.3	**0.005**	**<0.001**	**0.014**
Serum IL-6 (pg/ml)	1.5 ± 0.1	2.4 ± 0.2	4.1 ± 0.5	**0.006**	**<0.001**	**<0.001**
Serum IL-18 (pg/ml)	149 ± 18	235 ± 19	301 ± 23	**0.001**	**<0.001**	<0.05

Values are expressed as mean ± SEM. C-NMHE and C-MHE, cirrhotic patients without and with Minimal Hepatic Encephalopathy, respectively; ns, not significant; PHES, Psychometric Hepatic Encephalopathy Score; ^a^CVLT, Spanish adapted version of California Verbal Learning Test^48^. Child Pugh Score is derived from a score of 1–3 given for severity of ascites, hepatic encephalopathy, INR, albumin and bilirubin. The higher the score is, the more severe the liver disease. Differences between groups were analyzed using one-way ANOVA followed by post-hoc multiple comparisons Bonferroni test. Using Bonferroni correction for multiple comparisons (n = 3), *P* values < 0.016 were considered as significant (in bold).

**Figure 1:**
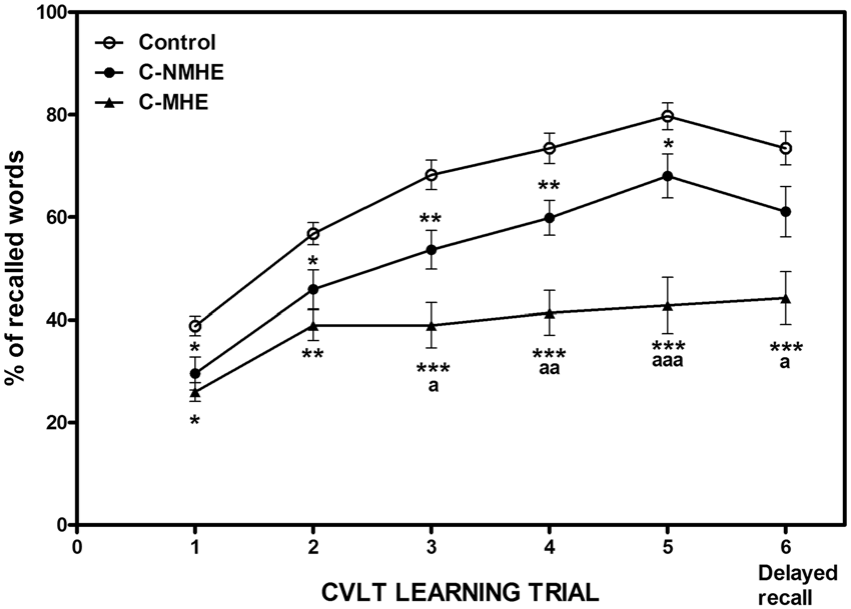
Comparison of percentages in recalled words in California Verbal Learning Test (CVLT) across the five learning trials in each experimental group. Trial 6 corresponds to percentage of words in delayed recall task. C-NMHE, cirrhotic patients without minimal hepatic encephalopathy; C-MHE, cirrhotic patients with minimal hepatic encephalopathy. Values significantly different from controls are indicated by asterisks. *p < 0.05; **p < 0.01; ***p < 0.001. Values significantly different in C-MHE compared to C-NMHE patients are indicated by ^a^p < 0.05; ^aa^p < 0.01; ^aaa^p < 0.001.

Figure 1 shows the percentage of recalled words across the five trials and on the delayed recall task. Controls and C-NMHE patients showed an increasing percentage of recalled words across the five trials, whereas C-MHE patients did not increase their percentage of words learned from trial 2 to 5. The C-MHE group recalled a significantly lower percentage of words (44%) than controls (73%, p < 0.001) and C-NMHE patients (63%, p < 0.05) after an interval of 20 min (delayed recall, trial 6, in Fig. 1).

However, the percentage of recalled words resembled the percentages of words learned during the five runs in each group (controls: learned 79.7%, recalled 73%; C-NMHE: learned 68%, recalled 63%; C-MHE: learned 43%, recalled 44%), with no significant differences between learned and recalled percentages.

Regarding the biochemical parameters, both C-NMHE and C-MHE patients showed increased levels of IL6, IL18 in serum, cGMP in plasma, and blood levels of ammonia, compared to controls. In addition, C-MHE patients showed higher levels of IL6 (p < 0.001) and IL18 (p < 0.05) in serum, and cGMP in plasma (p = 0.01) compared to C-NMHE (Table 1).

### Correlation analysis of memory performance, cognitive impairment, and biochemical parameters

Partial correlations with gender as a covariate in the whole group of participants indicated a relationship between learning and memory performance and cognitive impairment (assessed by PHES), as revealed by the high correlations found between the PHES score and total learning (r = 0.569; p < 0.001), delayed recall (r = 0.442; p < 0.001), and the recognition score (r = 0.519; p < 0.001).

Blood levels of several biochemical parameters correlated negatively with the CVLT scores in the whole group of participants: Total learning correlated with plasma cGMP (r = −0.36; p = 0.004), serum IL-6 (r = −0.43; p < 0.001) and IL-18 (r = −0.488; p < 0.001). Delayed recall correlated with serum IL-6 (r = −0.36; p = 0.004) and IL-18 (r = −0.445; p < 0.001). There was a weak correlation between the recognition task and serum IL-6 (r = −0.25; p = 0.046).

Intra-group correlations showed that, in C-NMHE patients, there were significant correlations between CVLT delayed recall and blood ammonia (r = 0.46; p = 0.02) and serum IL-18 levels (r = −0.42; p = 0.04). No significant intra-group correlations were found for controls and C-MHE patients.

### Hippocampal subfield volumes

**Figure 2:**
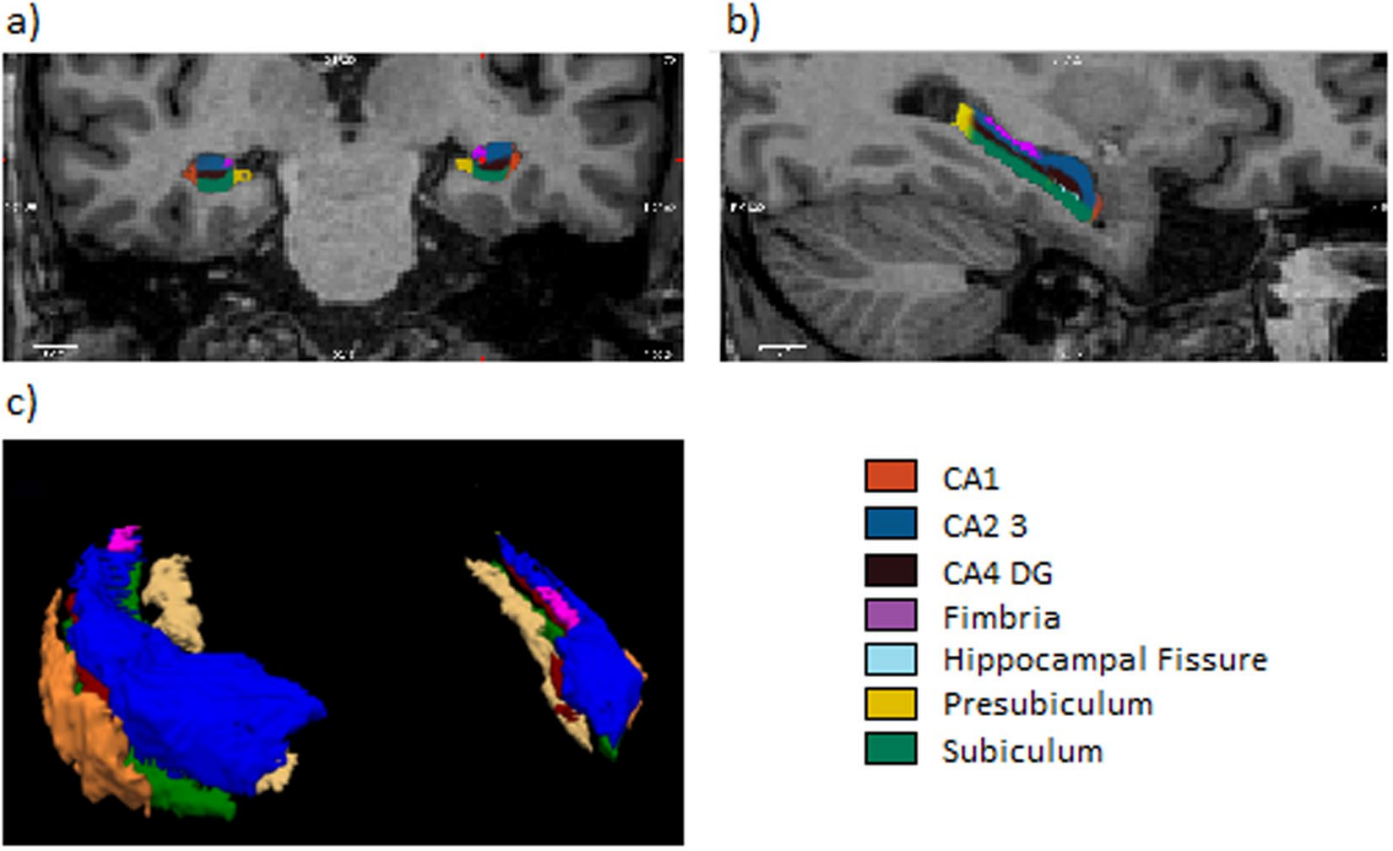
Hippocampal subfields segmentation in one subject. (**a**) Coronal and (**b**) axial slices of the hippocampal segmentation in detail. (**c**) 3D view of the binary hippocampal subfields regions of interest in MNI space.

Figure 2 shows the hippocampal subfield segmentation in one participant (Fig. 2a,b) and a 3D view of the binary hippocampal subfield regions of interest in the MNI space (Fig. 2c).

Regarding the hippocampal subfield analyses, only the volume of the right fimbria showed significant differences between the groups controlling, for gender. Specifically, both C-NMHE and C-MHE patients showed less volume than controls in this region (p < 0.05). Nonetheless, there were no differences between the two patient groups. No other significant differences were found in the other hippocampal regions (Table 2).

**Table 2:** Comparisons of hippocampal subfields volumes between controls and patients with or without MHE, and differences among groups in the resting-state functional connectivity of hippocampal subfields with precuneus.

Volume	Controls (n = 24)	C-NMHE patients (n = 25)	C-MHE patients (n = 13)	C-NMHE *P vs*. controls	C-MHE *P vs*. controls	C-MHE *P vs*. C-NMHE
**Left**						
Presubiculum	2.72 ± 0.09	2.59 ± 0.1	2.55 ± 0.21	0.513	0.597	0.998
CA1	1.89 ± 0.05	1.86 ± 0.07	1.98 ± 0.09	0.774	0.361	0.242
CA2-3	5.76 ± 0.23	5.60 ± 0.26	5.39 ± 0.34	0.704	0.515	0.726
Fimbria	0.38 ± 0.02	0.34 ± 0.03	0.33 ± 0.05	0.325	0.463	0.944
Subiculum	3.69 ± 0.14	3.54 ± 0.14	3.56 ± 0.24	0.662	0.988	0.715
CA4-DG	3.28 ± 0.13	3.18 ± 0.15	3.07 ± 0.19	0.730	0.541	0.734
Hippocampal Fissure	0.26 ± 0.02	0.29 ±0.03	0.34 ± 0.04	0.897	0.145	0.716
**Right**						
Presubiculum	2.62 ± 0.11	2.55 ± 0.09	2.45 ± 0.18	0.829	0.739	0.871
CA1	1.98 ± 0.07	2.00 ± 0.06	2.01 ± 0.11	0.764	0.562	0.732
CA2-3	6.13 ± 0.25	5.96 ± 0.23	5.91 ± 0.36	0.750	0.892	0.900
Fimbria	0.40 ± 0.02	0.31 ± 0.03	0.28 ± 0.04	**0.018**	**0.033**	0.836
Subiculum	3.69 ± 0.15	3.53 ± 0.12	3.54 ± 0.26	0.578	0.975	0.667
CA4-DG	3.44 ± 0.15	3.34 ± 0.13	3.28 ± 0.2	0.740	0.815	0.971
Hippocampal Fissure	0.31 ± 0.02	0.34 ± 0.03	0.37 ± 0.05	0.35	0.129	0.430
**Functional connectivity**	**Controls** **(n = 18)**	**C-NMHE patients (n = 19)**	**C-MHE patients (n = 10)**	**C-NMHE** ***P vs***. **controls**	**C-MHE** ***P vs***. **controls**	**C-MHE** ***P vs***. **C-NMHE**
***FC differences C-MHE*** < ***C-NMHE***						
L Presubiculum-L Precuneus	0.10 ± 0.04	0.13 ± 0.04	−0.16 ± 0.05	1.00	**0.001**	**<0.001**
***FC differences C-MHE*** < ***controls***						
L Subiculum-bilateral Precuneus	0.24 ± 0.04	0.10 ± 0.03	−0.004 ± 0.03	**0.005**	**<0.001**	0.068
L Presubiculum-bilateral Precuneus	0.24 ± 0.04	0.13 ± 0.03	−0.083 ± 0.04	0.116	**<0.001**	**0.003**

Hippocampal subfield volume measures (in mm^3^) are expressed as mean ± SEM, and were normalized by the subject’s intracranial volume (see Material and Methods section). Functional connectivity values are expressed as mean ± SEM of the eigenvalues obtained for each contrast. C-NMHE and C-MHE, cirrhotic patients without and with Minimal Hepatic Encephalopathy, respectively; CA: Cornu Ammonis; DG: Dentate Gyrus; L, left. Differences between groups were analyzed using General Lineal Model, with post-hoc multiple comparisons Bonferroni test, including gender as a nuisance variable. Significant *P* values (p < 0.05) are in bold.

### Correlation analysis of volume of hippocampal subfields, memory performance, and biochemical parameters

**Figure 3:**
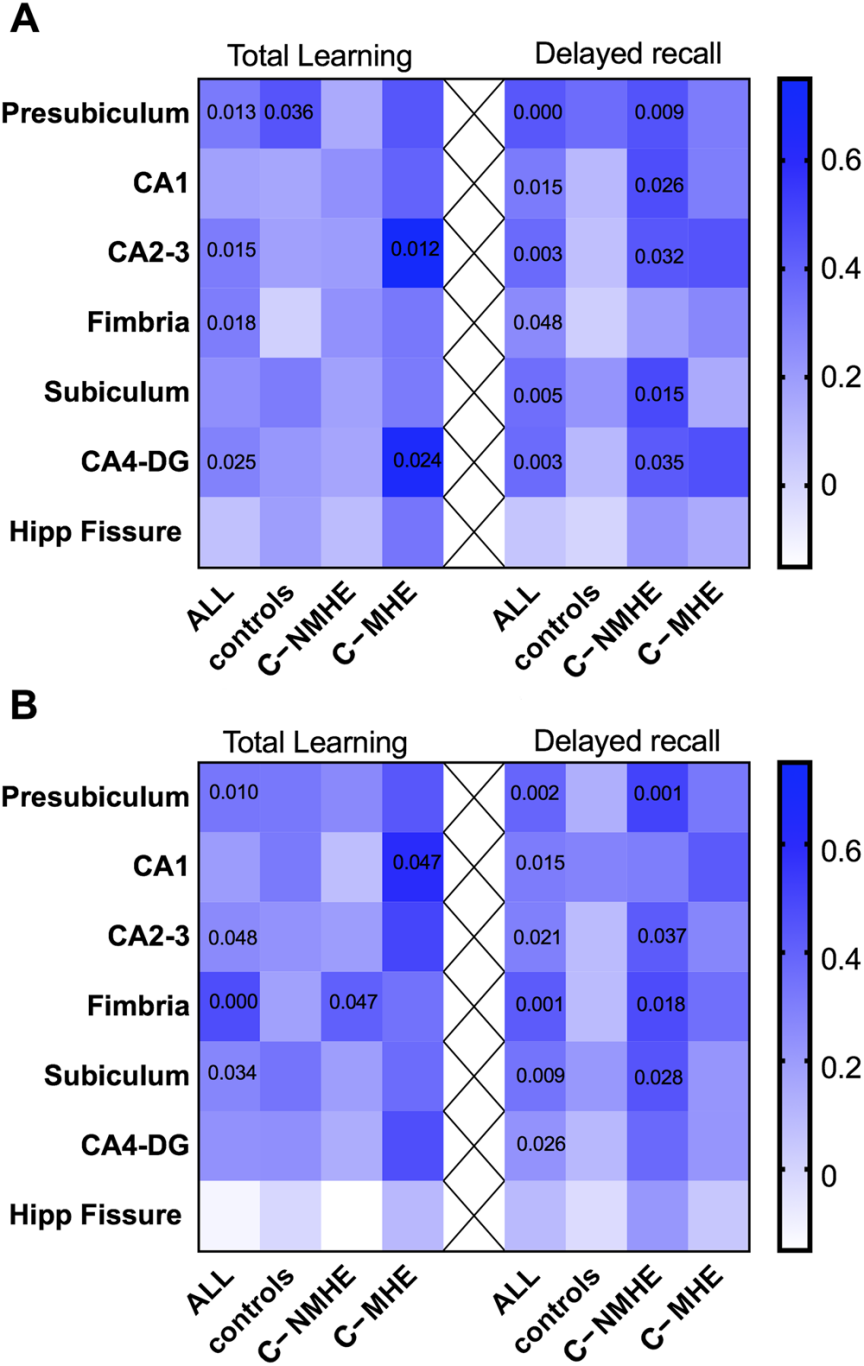
Correlational heat map depicting the relationship between different hippocampal subfield volumes of the left (**A**) and right (**B**) hemispheres and total learning and delayed recall in CVLT (California Verbal Learning Test). Separate correlations (columns) were calculated for the whole sample (ALL) and for each subgroup of participants: controls, cirrhotic patients without MHE (C-NMHE) and with MHE (C-MHE). Cells’ colour denotes Pearson’s r coefficients according to the graphical scale provided on the right side, while p values for statistically significant (p < 0.05) associations are provided within cells (the fully detailed output is provided on Supplementary Table S1). Note that, due to the different sample size, statistical power differs in each of these separate association analyses and there is not a strict correspondence between the colour scale and significance values (i.e. the same Pearson r coefficients might achieve statistical significance in larger groups but not in smaller ones).

Before proceeding to any correlational analyses, we searched for possible multivariate outliers using robust estimation of the parameters in the Mahalanobis distance and the comparison with the corresponding critical value of the chi-squared 2 distribution (for details, see Rousseeuw & Van Zomeren^38^, and Aguinis *et al*.^39^). Only one multivariate outlier case (from the MHE group) was identified and, therefore, excluded from subsequent correlational analyses. In the whole sample of participants, partial correlations with gender as covariate revealed that the volume of several hippocampal subregions of interest was clearly related to CVLT learning and delayed recall (Fig. 3 and Supplementary Table S1).

Intra-group correlations between volumes of hippocampal subregions showed that greater volume in different hippocampal subfields in C-NMHE patients positively correlated with better performance on CVLT delayed recall (Fig. 3 and Supplementary Table S1). Regarding the MHE patients, there were significant correlations between CVLT learning and the volume of the right CA1 and left CA2-3 and CA4-DG (Fig. 3 and Supplementary Table S1).

In addition, significant correlations were found between volumetric parameters and biochemical variables (see Supplementary Table S2 for further information). In the whole group of participants, levels of interleukin IL-18 in serum negatively correlated with the volume of some subregions of the hippocampus. Intra-group correlations showed that in the C-NMHE group, blood ammonia correlated positively, and IL-18 negatively, with the volume of several hippocampal subregions (e.g. with the fimbria) (Supplementary Table S2).

### Hippocampal subfield functional connectivity (FC) analysis

**Figure 4:**
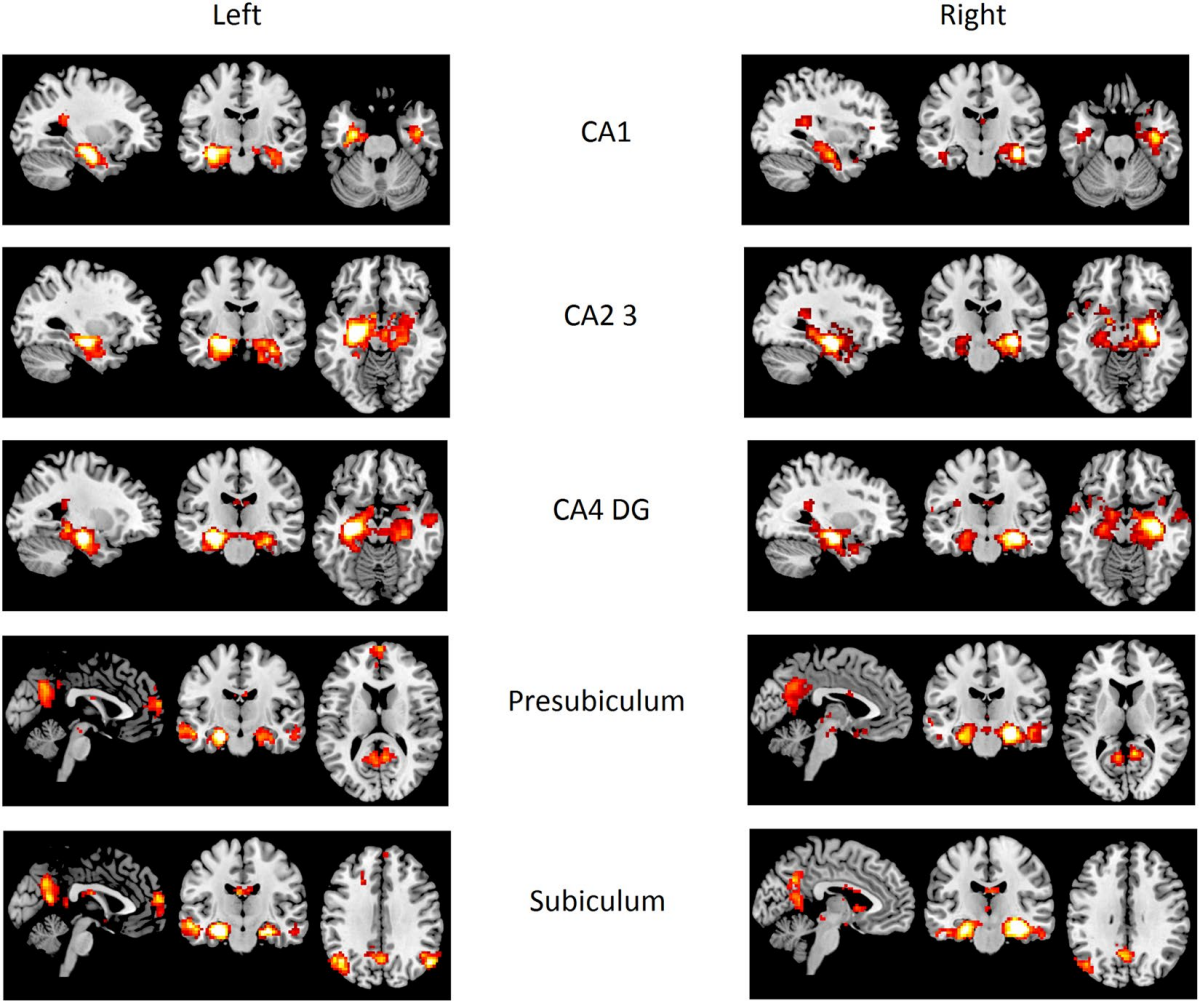
One sample tests in healthy controls representing functional connectivity voxel-wise maps connected to each hippocampal Region of Interest (ROI). CA, Cornu Ammonis; DG, Dentate Gyrus.

One sample tests for each group and each hippocampal ROI were performed in order to assess functionally connected brain regions. All the hippocampal seeds evoked FC maps that largely overlapped (see Fig. 4 for an illustrative example in the control group).

Due to excessive head motion in the fMRI, the FC was analyzed in fewer subjects (18 controls, 19 C-NMHE, and 10 C-MHE). The demographic, clinical, and neuropsychological data for these participants are shown in Supplementary Table S3. Similar results are obtained as in Table 1. The differences between C-MHE and C-NMHE patients in CVLT total learning remain significant; there is a trend toward significance (p = 0.06) on delayed recall when comparing C-MHE and C-NMHE patients, probably due to the lower number of patients in this sample, reaching significance when more number of patients are included, as shown in Table 1. Regarding hippocampal integrity, there is a trend toward a significant difference (p = 0.06) in the volume of the right fimbria in C-NMHE patients, compared to controls, in the group of participants whose FC was analyzed. In this group of participants, volumes of hippocampal subregions correlated with CVLT delayed recall, but no significant correlations were found with CVLT learning (Supplementary Table S4).

**Figure 5:**
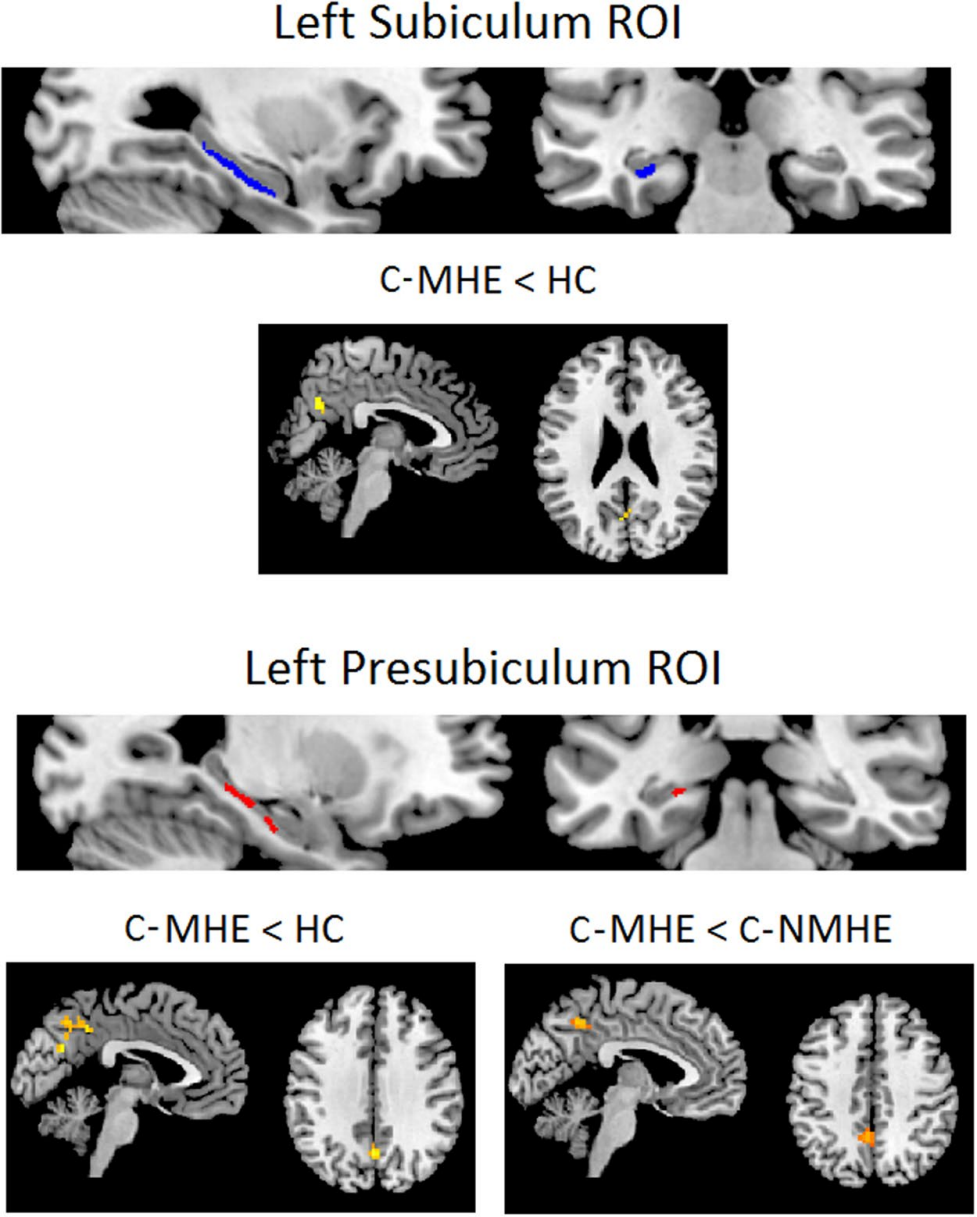
Significant differences in subiculum and presubiculum functional connectivity (FC) between groups in the ANCOVA design (including gender as nuisance covariate). Results were assessed at p < 0.05 FWE cluster-corrected for the multiple comparisons in a combination with a threshold of p < 0.001 at the uncorrected voxel level. ROI: Region of Interest; HC, healthy controls; C-NMHE, cirrhotic patients without minimal hepatic encephalopathy; C-MHE, cirrhotic patients with minimal hepatic encephalopathy. C-MHE < HC: C-MHE patients present a decreased FC in left subiculum and presubiculum with bilateral precuneus compared with controls. C-MHE < C-NMHE: C-MHE patients show decreased FC between the left presubiculum and left precuneus compared with C-NMHE patients.

Significant FC differences were observed between groups in the left subiculum and presubiculum seeds. More specifically, in both seeds, C-MHE patients exhibited less FC with the bilateral precuneus, compared to controls (cluster size = 36, MNI coordinates: −6–60 15; cluster size = 76, MNI coordinates: 0–63 33; see Fig. 5, Table 2). In addition, C-MHE patients also showed less FC between the left presubiculum and left precuneus compared to C-NMHE patients (cluster size = 28, MNI coordinates: −6–51 45; see Fig. 5, Table 2). The rest of the FC hippocampal subfield seeds showed no significant differences between groups.

### Correlational analyses of FC and memory performance, cognitive impairment, and biochemical parameters

Before proceeding with the correlational analyses, we searched for possible multivariate outliers using the same procedure described in the *hippocampal subfield volumes* section. In this case, no outliers were identified.

**Figure 6:**
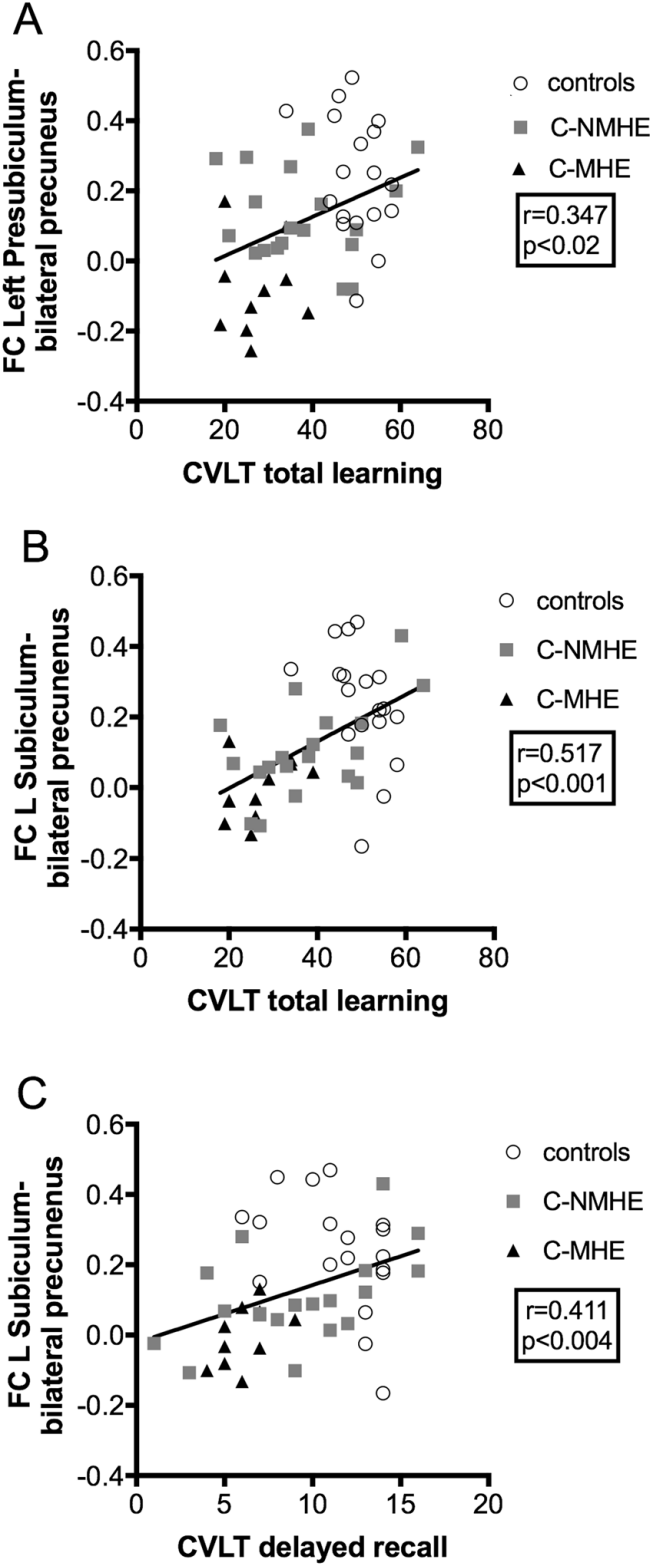
Correlations between CVLT parameters and functional connectivity (FC) in controls, cirrhotic patients without (C-NMHE) and with MHE (C-MHE); (**A**) positive correlations between CVLT total learning and FC between the left presubiculum and bilateral precuneus; (**B**) positive correlations between CVLT total learning and FC between left subiculum and bilateral precuneus; (**C**) positive correlations between CVLT delayed recall and FC between left subiculum and bilateral precuneus. CVLT, California Verbal Learning Test; MHE, minimal hepatic encephalopathy. The fully detailed output is provided on Supplementary Table S5.

In the entire sample of participants, partial correlations with gender as covariate revealed that FC in the subiculum and presubiculum seeds was significantly related to participants’ learning and delayed recall on the CVLT. Thus, CVLT total learning significantly correlated with FC between the left presubiculum and bilateral precuneus (r = 0.347, p < 0.02; Fig. 6A) and with FC between the left subiculum and bilateral precuneus (r = 0.536, p < 0.001; Fig. 6B). In addition, CVLT delayed recall also significantly correlated with the FC between the left subiculum and bilateral precuneus (r = 0.411, p < 0.004; Fig. 6C), and clear trends towards statistical significance were also found when considering the FC between the left presubiculum and bilateral precuneus (r = 0.248, p = 0.09) and between the left presubiculum and the left precuneus (r = 0.254, p = 0.08) (see Supplementary Table 5). The sign and direction of these correlations indicate that better performance on the CVLT subtest correlated with higher FC in the subiculum/presubiculum regions. Moreover, Fig. 6 reveals, the different subgroups of participants were ordered (control > C-NMHE > C-MHE) in these bivariate spaces.

In a second step, we sought to explore whether there were also significant intra-group correlations between the FC in these three hippocampal seeds and CVLT learning/delayed recall performance (Supplementary Table S5). Interestingly, only significant intra-group correlations were found between the left subiculum and bilateral precuneus. More specifically, we observed that FC connectivity in this seed was directly and significantly correlated with CVLT total learning in the C-NMHE group (r = 0.523, p = 0.03).

In a similar way, FC connectivity between the left subiculum and bilateral precuneus was also significantly and directly correlated with CVLT delayed recall in the C-NMHE group (r = 0.475, p < 0.05). A similar relationship was found for the C-MHE group (r = 0.564, p = 0.113), which, according to our power calculations, would have reached statistical significance if the number of participants in this group had been ≥17 instead of just the 10 in our study (Supplementary Table S5).

Moreover, in the whole sample of participants, FC connectivity in these three hippocampal seeds correlated with the PHES score, indicating an association between cognitive impairment and a loss of resting-state connectivity in these regions (Supplementary Table S5).

Correlations between FC parameters and biochemical variables in the whole sample of participants showed significant inverse correlations between FC in the left subiculum and bilateral precuneus and blood levels of cGMP (r = −0.318, p = 0.03) and IL-6 (r = −0.434, p = 0.003) and between FC in the left presubiculum and bilateral precuneus and IL-6 (r = −0.342, p = 0.02). No significant intra-group correlations were found between FC parameters and the biochemical variables. However, we found a significant correlation between FC in the left subiculum and bilateral precuneus and the volume of the right fimbria in the whole group of participants (r = 0.324; p = 0.028).

## Discussion

The present study describes episodic memory (learning and long-term memory) impairments in C-MHE and C-NMHE patients, and the neural correlates that underlie these deficits. To do so, we used a comprehensive combination of structural and functional methods, and we focused on the hippocampus, the area of the brain associated with episodic memory^40^.

There are no studies that evaluate the relationship between memory function in MHE and structural and functional connectivity (FC) changes in the hippocampal system.

Behavioral results showed poor performance on verbal learning and long-term memory (delayed recall) in cirrhotic patients (C-MHE and C-NMHE), compared to healthy controls. As expected, lower performance on episodic verbal memory was more apparent in C-MHE patients than in C-NMHE patients. These results agree with Torres *et al*.^41^, who described impairments in verbal episodic memory and information processing speed in C-MHE patients.

Regarding the volumetric analysis of the hippocampal subfields, we observed a decreased volume in the right fimbria in the two groups of patients, compared to the control group. Efferent axons of the pyramidal cells in the hippocampus emerge and converge to form the fimbria, a prominent band of white matter. The fimbria carries axons that emanate primarily from pyramidal neurons in the CA1 and subiculum^21^. Alterations in the output of hippocampus information due to alterations in the integrity of the fimbria could explain the patients’ reduced learning capacity and long-term memory of the patients. This proposal seems to be further reinforced by the observed direct correlations between the volume of different hippocampal subfields and CVLT performance (see Fig. 3). Of note, these correlations were more abundant and achieved higher correlation indexes for delayed recall than for total learning. Moreover, most of these correlations were stronger in patients than in the control group (see color shades in Fig. 3) although, due to statistical power limitations, only a few of them reached statistical significance in the C-MHE group.

FC analysis also revealed functional alterations between hippocampal subfields and cortical areas related to low memory performance. Regarding this specific analysis, reduced FC was observed in C-MHE patients, compared to healthy controls, between the left subiculum and presubiculum and bilateral precuneus. Furthermore, we also observed that C-MHE patients presented lower connectivity between the left presubiculum and left precuneus, compared to the C-NMHE patients. Once again, correlation analysis shows that reduced FC between these areas is significant in explaining memory performance in cirrhotic patients because, as Fig. 6 shows, decreased FC observed in both groups of patients is associated with worse performance on total learning and delayed recall.

We cannot overlook the attention problems observed in cirrhotic patients, a cognitive deficit that could interfere in episodic memory processes. In this regard, in a previous study, Weissenborn *et al*.^16^ concluded that lower memory performance in patients with early HE could be due to attention deficits that interfere with the encoding of the material to be memorized. The attention process is a basic function that interferes in the execution of other high cognitive processes, such as encoding and long-term memory storage. The process of cognitive control (as in attentional processes) is supported by neural networks such as the DMN (Default Mode Network), the salience network, and the LFPN (Left Frontoparietal Network). In a recent study^24^, we found that patients with MHE show reduced FC in these networks, and, specifically, we demonstrated that the most affected structure in the DMN is the precuneus. Consistent with previous results, the present study demonstrates the alteration of the FC between the hippocampal subregion L presubiculum and the precuneus in C-MHE patients, compared to C-NMHE patients and healthy controls. These areas are responsible for the formation and storage of memory and attention and executive control, respectively, all of them necessary processes for the correct execution of the CVLT.

Increased ammonia levels and inflammation contribute to neurological alterations in C-MHE patients^1,35–37^. In fact, hyperammonemia per se induces neuroinflammation, which plays a major role in neurological impairment in MHE^1^. In animal models of HE, hyperammonemia produces structural alterations in the entorhinal cortex^42^. In this study we aimed to determine whether these factors also contribute to the low performance on learning and memory found in these patients. Results showed that inflammatory variables are associated with the low performance on learning and long-term memory tasks in cirrhotic patients, given the inverse correlations found between the CVLT parameters and serum levels of interleukins IL-6 and IL-18. These results agree with a previous study in patients with HIV that related the levels of different plasma cytokines with memory performance in patients with HIV disease^43^. Moreover, as the intra-group correlations in C-NMHE patients show, a combination of increased ammonia and IL-18 blood levels could contribute to low performance on long-term memory. Although these patients did not show significantly lower delayed recall than controls, the intra-group correlations found could be explained by the fact that C-NMHE patients are a heterogeneous group. Some patients in this group showed low performance on memory and also low FC, and this would explain the correlations found in this group of patients. Moreover, we previously found that some patients classified as not having MHE (C-NMHE), based on the PHES score, showed attention and coordination deficits, and that the earliest neurological alterations vary in different cirrhotic patients. We also found that some of these patients have a high risk of suffering clinical complications, including overt HE, and they must be diagnosed and clinically followed^44^.

Finally, it is important to emphasize the growing evidence about the role of the parahippocampal cortex in episodic memory^45^. Unfortunately, the algorithm used in our study only includes subcortical structures, dividing the hippocampus into several hippocampal subfields, but leaving out related cortical structures such as the parahippocampal gyrus. Future studies would be needed to ascertain the possible role of parahippocampal cortical regions in the memory deficits of C-MHE patients. Another limitation is related to the segmentation protocol used in the present study, which only attends to the anterior-posterior axis when dividing the hippocampus into relevant subfields. However, as suggested by the results of some recent studies, the hippocampus might have a more complex functional organization based on longitudinal axis gradients^46^. Once again, future studies must be carried out to develop appropriate algorithms that may allow the automatic segmentation of the hippocampus along the longitudinal axis, and explore the relevance of the resulting hippocampal subfields and networks in episodic memory performance.

## Conclusion

This is one of the few studies addressing episodic memory deficits and their associated underlying neuropathology in cirrhotic patients. We observed that both C-MHE and C-NMHE patients presented learning and delayed memory impairments associated with functional and structural alterations in hippocampal structures. As expected, these behavioral and anatomical alterations are more prominent in C-MHE patients than in the C-NMHE group. FC may be more sensitive than structural analysis in detecting alterations in the hippocampal system associated with learning and memory in C-MHE patients. Although it is true that these patients present other types of cognitive deficits (eg, attention alterations), this study demonstrates that alterations in hippocampal zones make the learning process difficult, thus interfering with new information storage.

In cirrhotic patients, the combination of certain levels of hyperammonemia and inflammation is associated with cognitive impairment, not only in cognitive functions previously described as features of these patients, such as attention and coordination, but also in memory and learning processes related to structural and functional hippocampal dysfunctions.

Nonetheless, future studies must be conducted to strengthen the results of this study.

## Materials and Methods

### Participants

Thirty-eight patients with liver cirrhosis and 24 healthy controls without liver disease were recruited after giving their written informed consent. Patients were from the outpatients clinics at the Clinico Universitario and Arnau de Vilanova Hospitals, in Valencia, and they were included if they had clinical, biochemical, and histological evidence of liver cirrhosis. Exclusion criteria were overt HE or history of overt HE, recent (<6 months) alcohol intake, infection, recent (<6 weeks) antibiotic use or gastrointestinal bleeding, use of drugs affecting cognitive function, hepatocellular carcinoma, or neurological or psychiatric disorders. In the case of controls, liver disease was ruled out through clinical, analytical, and serologic analyses. All the participants were volunteers.

Neuropsychological measures and blood collection were carried out on the same day. Cerebral fMRI was performed in the week following the neuropsychological assessment. Study protocols were approved by the Scientific and Ethical Committees of the Clínico and Arnau de Vilanova Hospitals, Valencia, Spain, and they were in accordance with the ethical guidelines of the Helsinki Declaration.

### Neuropsychological measures

Patients were classified as being without MHE (C-NMHE) (25 patients) or with MHE (C-MHE) (13 patients), according to the Psychometric Hepatic Encephalopathy Score (PHES), which includes five subtests: Digit Symbol test (DST), number connection tests A and B (NCT-A and NCT-B), Serial Dotting test (SDT), and Line Tracing test (LTT)^2,7^. The PHES score was calculated, adjusting for age and educational level, using Spanish normality tables (www.redeh.org). Patients were classified as C-MHE when the score was ≤ −4 points^7^.

In addition, learning and long-term memory were assessed with the Test de Aprendizaje Verbal España Complutense (TAVEC)^47^, the Spanish adapted version of the California Verbal Learning Test (CVLT)^48^. The CVLT is a test of verbal memory involving immediate recall of a 16-item word list. In the immediate recall phase, the examiner reads the list of words that have to be repeated by the subject. Five trials are administered. After a 20 min interval, the subject is asked to remember the words learned before. This free recall trial is followed by a recognition recall trial. Scores used in this analysis are: (1) total learning: total number of recalled words across the five learning trials; (2) delayed recall: the number of words recalled after the 20-min delay; and (3) Recognition: number of words on the recognition recall trial.

We classified patients with liver cirrhosis into patients with or without MHE, according to the PHES score, with the aim of detecting differences in learning, memory performance, and the structural integrity and functional connectivity of the hippocampal system between the two groups of patients. We previously found that patients who do not show MHE on the PHES (i.e. patients classified as “without MHE”) already have some mild neurological alterations that are not detected with the PHES^44^. Therefore, we also included a group of healthy controls in our study in order to detect possible differences in learning and memory performance between C-NMHE patients and healthy controls.

### Biochemical determinations in blood

Interleukins IL-6 and IL-18 were measured in serum using Human IL-6 and IL-18 Platinum ELISA kits from eBioscience (Bender MedSystems GmbH, Vienna, Austria). Blood ammonia was measured immediately after blood collection with the Ammonia Test Kit II for the PocketChemBA system (Arkay, Inc., Kyoto, Japan). Cyclic GMP (cGMP) in plasma was determined using the BIOTRAK cGMP enzyme immunoassay kit from Amersham (GE Healthcare, Life Sciences, UK).

### MRI acquisition

All subjects underwent an MRI scan using a 3 T Philips Achieva scanner (Philips Medical Systems, Netherlands) in the week following the neuropsychological assessment. Sagittal high-resolution three-dimensional 3D MPRAGE T1 images were acquired (TR = 8.42 ms, TE = 3.8 ms, matrix = 320 × 320 × 250, voxel size = 1 × 1 × 1 mm, flip angle = 8°). Furthermore, the fMRI resting-state sequence was also acquired using a gradient-echo T2*-weighted echo-planar imaging (EPI) sequence (5 min, 150 volumes, TR = 2000ms, TE = 30 ms, matrix = 80 × 80 × 31, voxel size = 3 × 3 × 3 mm, flip angle = 85°). During this sequence, subjects were instructed to keep their eyes open, remain motionless, relax and not think of anything in particular.

### Hippocampal subfield volumes

Anatomical T1-weighted images were processed by Freesurfer 5.3, a fully automated image analysis software for the volumetric segmentation of hippocampal subfields (http://surfer.nmr.mgh.harvard.edu). Specific details of this procedure are available in previous publications^49^. Briefly, this procedure includes motion correction, removal of non-brain, automated Talairach transformation, segmentation of the subcortical white matter and deep gray matter structures, intensity normalization, tessellation of the gray matter/white matter boundary, automated topology correction, and surface deformation following gradients to place the gray/white and gray/cerebrospinal fluid borders optimally at the location where the greatest shift in intensity defines the transition to the other tissue type. Subcortical segmentation is achieved by aligning the target image with an atlas constructed from a set of labeled training images.

Subsequently, the hippocampal subfield segmentation automatic reconstruction command was included. This procedure uses Bayesian inference and a probabilistic atlas of the hippocampal formation, which is based on manual segmentations of the subfields in T1-weighted MRI scans from different subjects^50^. The process also segments both the hippocampi of each subject into eight subfields, including the cornu ammonis 1 (CA1), cornu ammonis 2–3 (CA2-3), cornu ammonis 4-dentade gyrus (CA4-DG), subiculum, presubiculum, fimbria, hippocampal fissure, and posterior hippocampus. The technical details of these procedures have been described elsewhere^49^.

Two outputs were obtained from this analysis: (1) a probabilistic ROI for each subject for each hippocampal subfield in native space (see Fig. 2a,b) and (2) estimated volumes for each subject and each subfield in mm^3^. Hippocampal subfield volume measures were normalized by the subject’s intracranial volume (ICV), derived from Freesurfer with the following formula: volume_norm_ = volume_raw_ × 1000/ICV in cm^3^.

We applied the same procedure for hippocampal subfield segmentation with the “ch2” template included in MRIcron (www.sph.sc.edu/comd/rorden/mricron) to obtain the same probabilistic ROI for each hippocampal structure in standard MNI space. Each probability map was binarized with IMcalc from SPM12 at a threshold of 95%. Each binary mask was used as a functional connectivity (FC) seed in the subsequent analyses (see Fig. 2c).

### Resting state functional connectivity (FC) analysis

Resting-state fMRI images were pre-processed using the DPARSF V4.3 tool^51^ and included: discarding the first 10 functional volumes to reach a signal equilibrium, slice timing correction, realignment to the first scan of each session, head motion correction, coregister, nuisance covariate regression to remove nonspecific sources of variance (including 24-parameter head motion model, white matter signal, CSF and global signal), spatial normalization with a resampled voxel size of 3 mm^3^ to the Montreal Neurological Institute (MNI) space, and spatial smoothing with an isotropic Gaussian kernel of 4-mm full width at half maximum (FWHM). Lastly, temporal filtering (0.01 Hz–0.08 Hz) was applied to the time series of each voxel to reduce the effect of low-frequency drifts and high-frequency noise. After these steps, 9 patients (6 C-NMHE and 3 C-MHE) and 6 controls were excluded due to excessive head motion (>1.5 mm translation, >1.5degrees of angular motion, or >0.5 mm mean frame-wise displacement).

Twelve ROIs corresponding to bilateral CA1, CA2-3, CA4-DG, Fimbria, Presubiculum, and Subiculum in MNI space were used as seeds in subsequent FC analyses, averaging the time series from each ROI and correlating with the time series of every voxel in the brain. Hippocampal fissure and fimbria ROIs were discarded due to their non-brain gray matter tissue localization.

### Statistical analysis

Statistical analysis was performed using SPSS (Version 22.0, Chicago, IL). Demographic, clinical, and neuropsychological variables of the groups were compared using one-way ANOVAs and Bonferroni’s post hoc test was used to analyze differences among the three groups. The groups’ hippocampal subfield volumes were compared following the General Lineal Model. Each subfield volume was included as a dependent variable, subgroup was included as a factor with three levels (controls, C-NMHE and C-MHE), and gender was included as a nuisance variable.

The FC of the hippocampal subfield seeds was assessed using SPM12. First, one sample tests were performed in each group to disentangle voxel-wise maps evoked by each ROI. FC differences between groups were then assessed using an analysis of covariance (ANCOVA) in SPM12, including gender as a nuisance variable. Results were assessed using an implicit mask of the union of the one-samples corresponding to each experimental group in each seed with p < 0.05 FWE, cluster-corrected for the multiple comparisons in a combination, with a threshold of p < 0.001 at the uncorrected level.

Mean signal intensity values (SPM eigenvalues) of significant difference between FC clusters were extracted and correlated with the neuropsychological and clinical variables (p < 0.05).

### Data availability statement

All data generated or analyzed during this study are included in this published article.

## Electronic supplementary material

Supplementary Tables
