# Supplementary material for: Learning and Memory Impairments in Patients with Minimal Hepatic Encephalopathy are Associated with Structural and Functional Connectivity Alterations in Hippocampus
Source: Sci Rep. 2018 Jun 25;8:9664. doi: 10.1038/s41598-018-27978-x (PMC6018225; doi:10.1038/s41598-018-27978-x)
Supplement: Supplementary file 1 — Supplementary Tables [file 41598_2018_27978_MOESM1_ESM.pdf]

LEARNING AND MEMORY IMPAIRMENTS IN PATIENTS WITH MINIMAL HEPATIC ENCEPHALOPATHY ARE ASSOCIATED WITH STRUCTURAL AND FUNCTIONAL CONNECTIVITY ALTERATIONS IN HIPPOCAMPUS

Raquel García-García, Álvaro Javier Cruz-Gómez, Amparo Urios, Alba Mangas-Losada, Cristina Forn, Desamparados Escudero-García, Elena Kosenko, Isidro Torregrosa, Joan Tosca, Remedios Giner-Durán, Miguel Angel Serra, César Avila, Vicente Belloch, Vicente Felipo and Carmina Montoliu

SUPPLEMENTARY TABLES

- **Supplementary Table S1.** Correlations between volumes of hippocampal regions and CVLT parameters in all the groups studied.
- **Supplementary Table S2.** Significant correlations between volumes of hippocampal regions and biochemical markers in all the groups studied.
- **Supplementary Table S3.** Main demographic, clinical and neuropsychological characteristics of participants in whom functional connectivity was analyzed.
- **Supplementary Table S4.** Correlations between volumes of hippocampal regions and CVLT parameters in participants in whom functional connectivity was analyzed (18 controls, 19 C-NMHE and 10 C-MHE).
- **Supplementary Table S5.** Significant correlations between functional connectivity of hippocampal seeds and neuropsychological measures in patients.

		Left hippocampal areas						
		Presubiculum	CA1	CA2-3	Fimbria	Subiculum	CA4-DG	Hippocampal Fissure
**CVLT total learning**	**All participants**	**r=0.319**<br>p=0.013	r=0.181<br>p=0.167	**r=0.312**<br>**p=0.015**	**r=0.306**<br>**p=0.018**	r=0.241<br>p=0.064	**r=0.290**<br>**p=0.025**	r= -0.066<br>p=0.615
	**Controls**	**r=0.438**<br>**p=0.036**	r=0.161<br>p=0.463	r=0.185<br>p=0.398	r=0.014<br>p=0.950	r=0.311<br>p=0.148	r=0.214<br>p=0.327	r=0.191<br>p=0.382
	**C-NMHE patients**	r=0.143<br>p=0.505	r=0.235<br>p=0.268	r=0.197<br>p=0.357	r=0.233<br>p=0.272	r=0.179<br>p=0.403	r=0.167<br>p=0.435	r=-0.085<br>p=0.694
	**C-MHE patients**	r=0.443<br>p=0.172	r=0.399<br>p=0.224	**r=0.724**<br>**p=0.012**	r=0.324<br>p=0.331	r=0.314<br>p=0.347	**r=0.670**<br>**p=0.024**	r=0.331<br>p=0.320
**CVLT Delayed recall**	**All participants**	**r=0.438**<br>**p=0.000**	**r=0.313**<br>**p=0.015**	**r=0.375**<br>**p=0.003**	**r=0.257**<br>**p=0.048**	**r=0.360**<br>**p=0.005**	**r=0.372**<br>**p=0.003**	r=0.053<br>p=0.690
	**Controls**	r=0.364<br>p=0.088	r=0.094<br>p=0.670	r=0.073<br>p=0.739	r=0.022<br>p=0.922	r=0.223<br>p=0.307	r=0.099<br>p=0.653	r=- 0.001<br>p=0.996
	**C-NMHE patients**	**r=0.454**<br>**p=0.026**	**r=0.478**<br>**p=0.018**	**r=0.439**<br>**p=0.032**	r=0.189<br>p=0.378	**r=0.491**<br>**p=0.015**	**r=0.431**<br>**p=0.035**	r=0.218<br>p=0.306
	**C-MHE patients**	r=0.311<br>p=0.352	r=0.303<br>p=0.365	r=0.451<br>p=0.164	r=0.269<br>p=0.423	r=0.144<br>p=0.673	r=0.446<br>p=0.170	r=0.144<br>p=0.672

		Right hippocampal areas						
		Presubiculum	CA1	CA2-3	Fimbria	Subiculum	CA4-DG	Hippocampal Fissure
**CVLT total learning**	**All participants**	r=0.329<br>p=0.10	r=0.197<br>p=0.131	**r=0.257**<br>**p=0.048**	**r=0.478**<br>**p=0.000**	**r=0.274**<br>**p=0.034**	r=0.233<br>p=0.074	r= -0.117<br>p=0.374
	**Controls**	r=0.323<br>p=0.133	r=0.319<br>p=0.138	r=0.229<br>p=0.293	r=0.180<br>p=0.412	r=0.335<br>p=0.118	r=0.239<br>p=0.272	r=-0.011<br>p=0.961
	**C-NMHE patients**	r=0.265<br>p=0.211	r=0.077<br>p=0.722	r=0.195<br>p=0.361	r=0.410<br>p=0.047	r=0.192<br>p=0.370	r=0.136<br>p=0.526	r=-0.150<br>p=0.483
	**C-MHE patients**	r=0.437<br>p=0.179	**r=0.609**<br>**p=0.047**	r=0.566<br>p=0.070	r=0.345<br>p=0.298	r=0.367<br>p=0.267	r=0.474<br>p=0.141	r=0.097<br>p=0.778
**CVLT Delayed recall**	**All participants**	**r=0.390**<br>**p=0.002**	**r=0.312**<br>**p=0.015**	**r=0.296**<br>**p=0.021**	**r=0.428**<br>**p=0.001**	**r=0.337**<br>**p=0.009**	**r=0.288**<br>**p=0.026**	r=0.093<br>p=0.482
	**Controls**	r=0.129<br>p=0.557	r=0.286<br>p=0.186	r=0.087<br>p=0.692	r=0.089<br>p=0.687	r=0.213<br>p=0.329	r=0.100<br>p=0.651	r=-0.028<br>p=0.900
	**C-NMHE patients**	**r=0.513**<br>**p=0.010**	r=0.305<br>p=0.147	**r=0.428**<br>**p=0.037**	**r=0.479**<br>**p=0.018**	**r=0.449**<br>**p=0.028**	r=0.379<br>p=0.068	r=0.214<br>p=0.316
	**C-MHE patients**	r=0.324<br>p=0.331	r=0.430<br>p=0.187	r=0.274<br>p=0.415	r=0.356<br>p=0.282	r=0.218<br>p=0.519	r=0.218<br>p=0.519	r=0.046<br>p=0.894

Supplementary Table S1. Correlations between volumes of hippocampal regions and CVLT parameters in all the groups studied. Data show values of partial correlations, adjusting for gender. CA, Cornu Ammonis; CVLT, California Verbal Learning Test; DG: Dentate Gyrus; L, left; C-NMHE, C-MHE, cirrhotic patients without or with minimal hepatic encephalopathy, respectively; Post, posterior; R, right; ns, non-significant correlations. Significant correlations ($p<0.05$) are in bold.

	All participants		Controls		C-NMHE		C-MHE	
Hippocampal regions	Ammonia	IL-18	Ammonia	IL-18	Ammonia	IL-18	Ammonia	IL-18
Left hippocampal areas								
L Presubiculum	ns	**r=-0.27 p=0.04**	ns	ns	ns	**r=-0.545 p=0.006**	ns	ns
L CA1	**r=0.36 p=0.004**	ns	ns	ns	**r=0.501 p=0.013**	r=-0.40 p=0.05	ns	ns
L CA2-3	ns	**r=-0.298 p=0.02**	ns	ns	ns	**r=-0.42 p=0.04**	ns	ns
L Fimbria	ns	**r=-0.3 p=0.02**	ns	ns	**r=0.53 p=0.008**	**r=-0.41 p=0.04**	ns	ns
L Subiculum	ns		ns	ns	ns	**r=-0.45 p=0.03**	ns	ns
L CA4-DG	ns	**r=-0.288 p=0.02**	ns	ns	**r=0.415 p=0.04**	**r=-0.44 p=0.03**	ns	ns
L Hippocampal fissure	ns	ns	ns	ns	ns	ns	ns	ns
Right hippocampal areas								
R Presubiculum	ns	ns	ns	ns	ns	**r=-0.46 p=0.02**	ns	ns
R CA1	ns	ns	ns	ns	**r=0.44 p=0.03**	r=-0.399 p=0.054	ns	ns
R CA2-3	ns	ns	ns	ns	ns	**r=-0.42 p=0.04**	ns	ns
R Fimbria	ns	**r=-0.32 p=0.012**	ns	ns	**r=0.47 p=0.02**	r=-0.37 p=0.07	ns	ns
R Subiculum	ns	ns	ns	ns	ns	**r=-0.5 p=0.013**	ns	ns
R CA4-DG	ns	ns	ns	ns	ns	**r=-0.48 p=0.02**	ns	ns
R Hippocampal fissure	ns	ns	ns	ns	ns	ns	ns	ns

Supplementary Table S2. Significant correlations between volumes of hippocampal regions and biochemical markers in all the groups studied. Data show values of partial correlations adjusting for gender. CA, Cornu Ammonis; CVLT, California Verbal Learning Test; DG: Dentate Gyrus; L, left; C-NMHE, C-MHE, cirrhotic patients without or with minimal hepatic encephalopathy, respectively; Post, posterior; R, right; ns, non-significant correlations. Significant correlations ($p<0.05$) are in bold.

	Controls (n=18)	C-NMHE patients (n=19)	C-MHE patients (n=10)	C-NMHE *P vs.* controls	C-MHE *P vs.* controls	C-MHE *P vs.* C-NMHE
**Age (range)**	60 ± 1 (50-73)	63 ± 2 (50-81)	65 ± 4 (49-85)	ns	ns	ns
**Gender (Male/Female)**	10 / 8	14 / 5	9 / 1			
**Child Pugh A/B/C**	-	17 / 2 / 0	7 / 3/ 0			
**Alcohol / HCV/ others**	-	7 / 10/ 2	4 / 3 / 3			
**PHES score**	1 ± 0.2	-1.1 ± 0.3	-7.5 ± 1.1	**0.005**	**<0.001**	**<0.001**
**CVLT**[a]						
Learning trial 1	6.2 ± 0.3	3.9 ± 0.5	3.9 ± 0.3	**<0.001**	**0.004**	1.000
Learning trial 2	8.9 ±0.4	6.7 ± 0.7	5.8 ± 0.3	0.019	**0.005**	1.000
Learning trial 3	10.8 ± 0.5	7.8 ± 0.6	5.6 ± 0.6	**0.002**	**<0.001**	0.07
Learning trial 4	11.7 ± 0.5	9.1 ± 0.6	6.1 ± 0.6	**0.007**	**<0.001**	**0.008**
Learning trial 5	12.3 ± 0.5	10.3 ± 0.8	5.8 ± 0.8	0.12	**<0.001**	**0.001**
Total Learning	50 ± 1	38 ± 3	27 ± 2	**0.001**	**<0.001**	**0.017**
Delayed Recall	11.4 ± 0.6	9.2 ± 0.9	6.1 ± 0.4	0.15	**0.001**	0.06
Recognition	14.7 ± 0.4	14.9 ± 0.3	12.9 ± 0.6	1.000	0.026	**0.009**
Biochemical determinations						
**Blood ammonia (μM)**	9.5 ± 0.8	20 ± 5	32 ± 7	0.285	**0.011**	1.000
**Plasma cGMP (pmol/ml)**	4.2 ± 0.2	8 ± 1.2	13 ± 1.4	**0.015**	**<0.001**	**0.008**
**Serum IL-6 (pg/ml)**	1.5 ± 0.1	2.6 ±0.2	4.1 ± 0.6	**0.005**	**<0.001**	**0.001**
**Serum IL-18 (pg/ml)**	145 ± 19	273 ± 23	321 ± 26	**<0.001**	**<0.001**	0.570

Supplementary Table S3. Main demographic, clinical and neuropsychological characteristics of participants in whom functional connectivity was analyzed.

Values are expressed as mean ± SEM. C-NMHE and C-MHE, cirrhotic patients without and with Minimal Hepatic Encephalopathy, respectively; PHES, Psychometric Hepatic Encephalopathy Score; [a]CVLT, Spanish adapted version of California Verbal Learning Test. Child Pugh Score is derived from a score of 1–3 given for severity of ascites, hepatic encephalopathy, INR, albumin, and bilirubin. The higher the score, the more severe the liver disease. Differences between groups were analyzed using one-way ANOVA followed by a post-hoc multiple comparisons Bonferroni test. Using Bonferroni correction for multiple comparisons (n=3), *P* values <0.016 were considered significant (in bold).

		Left hippocampal areas						
		Presubiculum	CA1	CA2-3	Fimbria	Subiculum	CA4-DG	Hippocampal Fissure
**CVLT total learning**	**All participants**	ns	ns	ns	ns	ns	ns	ns
	**Controls**	**ns**	**ns**	**ns**	**ns**	**ns**	**ns**	**ns**
	**C-NMHE patients**	**ns**	**ns**	**ns**	**ns**	**ns**	**ns**	**ns**
	**C-MHE patients**	**ns**	**ns**	**ns**	**ns**	**ns**	**ns**	**ns**
**CVLT Delayed recall**	**All participants**	**r=0.38 p=0.009**	**r=0.315 p=0.03**	**r=0.365 p=0.015**	ns	**r=0.338 p=0.02**	**r=0.363 p=0.013**	ns
	**Controls**	**ns**	**ns**	**ns**	**ns**	**ns**	**ns**	**ns**
	**C-NMHE patients**	**r=0.563 p=0.015**	**r=0.499 p=0.035**	r=0.464 p=0.052	**ns**	**r=0.585 p=0.01**	**r=0.473 p=0.048**	**ns**
	**C-MHE patients**	**ns**	**ns**	**ns**	**ns**	**ns**	**ns**	**ns**

		Right hippocampal areas						
		Presubiculum	CA1	CA2-3	Fimbria	Subiculum	CA4-DG	Hippocampal Fissure
**CVLT total learning**	**All participants**	**ns**	**ns**	**ns**	**ns**	**ns**	**ns**	**ns**
	**Controls**	**ns**	**ns**	**ns**	**ns**	**ns**	**ns**	**ns**
	**C-NMHE patients**	**ns**	**ns**	**ns**	**ns**	**ns**	**ns**	**ns**
	**C-MHE patients**	**ns**	**ns**	**ns**	**ns**	**ns**	**ns**	**ns**
**CVLT Delayed recall**	**All participants**	**r=0.350 p=0.02**	**ns**	**r=0.31 p=0.04**	**r=0.322 p=0.03**	r=0.282 p=0.058	**r=0.316 p=0.03**	**ns**
	**Controls**	**ns**	**ns**	**ns**	**ns**	**ns**	**ns**	**ns**
	**C-NMHE patients**	**r=0.639 p=0.004**	**ns**	**r=0.481 p=0.04**	**r=0.536 p=0.02**	**r=0.523 p=0.03**	**ns**	**ns**
	**C-MHE patients**	**ns**	**ns**	**ns**	**ns**	**ns**	**ns**	**ns**

Supplementary Table S4. Correlations between volumes of hippocampal regions and CVLT parameters in participants in whom functional connectivity was analyzed (18 controls, 19 C-NMHE and 10 C-MHE). Data show values of partial correlations adjusting for gender. CA, Cornu Ammonis; CVLT, California Verbal Learning Test; DG: Dentate Gyrus; L, left; NMHE, patients without minimal hepatic encephalopathy; Post, posterior; R, right. Significant correlations ($p<0.05$) are in bold.

		FC differences C-MHE<C-NMHE	*FC differences C-MHE<HC*	
		L Presubiculum-L Precuneus	L Subiculum-bilateral PC	L Presubiculum-bilateral PC
**PHES**	**All participants**	**r=0.438 p=0.002**	**r=0.415 p=0.004**	**r=0.458 p=0.001**
	**Controls**	ns	ns	ns
	**C-NMHE patients**	ns	**ns**	ns
	**C-MHE patients**	ns	**ns**	ns
CVLT-Total learning	**All participants**	r=0.210 p=0.161	**r=0.517 p<0.001**	**r=0.347 p=0.018**
	**Controls**	ns	**ns**	ns
	**C-NMHE patients**	ns	**r=0.523 p=0.026**	ns
	**C-MHE patients**	ns	ns	ns
CVLT-Delayed recall	**All participants**	r=0.254 p=0.088	**r=0.411 p=0.004**	r=0.248 p=0.096
	**Controls**	ns	**ns**	ns
	**C-NMHE patients**	ns	**r=0.475 p=0.046**	ns
	**C-MHE patients**	ns	r=0.564 p=0.113	ns
CVLT-Recognition	**All participants**	ns	ns	ns
	**Controls**	ns	**ns**	ns
	**C-NMHE patients**	ns	**ns**	ns
	**C-MHE patients**	ns	r=0.657 p=0.05	ns

Supplementary Table S5. Significant correlations between functional connectivity of hippocampal seeds and neuropsychological measures in patients. Data show values of partial correlations adjusting for gender. CVLT, California Verbal Learning Test; FC, functional connectivity; HC, healthy controls; L, left; C-NMHE, patients without minimal hepatic encephalopathy; C-MHE, patients with minimal hepatic encephalopathy; PC, precuneus; ns, non-significant correlations. Significant correlations ($p<0.05$) are in bold.
